# The viral capsid as novel nanomaterials for drug delivery

**DOI:** 10.2144/fsoa-2021-0031

**Published:** 2021-07-14

**Authors:** Alaa AA Aljabali, Sk Sarif Hassan, Ritesh M Pabari, Seyed H Shahcheraghi, Vijay Mishra, Nitin B Charbe, Dinesh K Chellappan, Harish Dureja, Gaurav Gupta, Abdulmajeed G Almutary, Abdullah M Alnuqaydan, Suresh K Verma, Pritam K Panda, Yogendra Kumar Mishra, Ángel Serrano-Aroca, Kamal Dua, Vladimir N Uversky, Elrashdy M Redwan, Bojlul Bahar, Amit Bhatia, Poonam Negi, Rohit Goyal, Paul McCarron, Hamid A Bakshi, Murtaza M Tambuwala

**Affiliations:** 1 Faculty of Pharmacy, Department of Pharmaceutics & Pharmaceutical Technology, Yarmouk University, Irbid, 21163, Jordan; 2 Department of Mathematics, Pingla Thana Mahavidyalaya, Paschim Medinipur, India; 3 School of Pharmacy, Royal College of Surgeons in Ireland, Dublin, Ireland; 4 Infectious Diseases Research Center, Shahid Sadoughi Hospital, Shahid Sadoughi University of Medical Sciences, Yazd, Iran; 5 School of Pharmaceutical Sciences, Lovely Professional University, Phagwara, Punjab, 144411, India; 6 Department of Pharmaceutical Sciences, Rangel College of Pharmacy, Texas A&M University, Kingsville, TX 78363, USA; 7 Department of Life Sciences, School of Pharmacy, International Medical University, Bukit Jalil, Kuala Lumpur, 57000, Malaysia; 8 Department of Pharmaceutical Sciences, Maharshi Dayanand University, Rohtak, 124001, India; 9 School of Pharmacy, Suresh Gyan Vihar University, Mahal Road, Jagatpura, Jaipur, 302017, India; 10 Department of Medical Biotechnology, College of Applied Medical Sciences, Qassim University, Saudi Arabia; 11 Condensed Matter Theory Group, Materials Theory Division, Department of Physics & Astronomy, Uppsala University, Uppsala, 75120, Sweden; 12 University of Southern Denmark, Mads Clausen Institute, NanoSYD, Alsion 2, Sønderborg 6400, Denmark; 13 Biomaterials & Bioengineering Lab, Centro de Investigación Traslacional San Alberto Magno, Universidad Católica de Valencia San Vicente Mártir, Valencia, 46001, Spain; 14 Discipline of Pharmacy, Graduate School of Health, University of Technology, Sydney, Australia; 15 Department of Molecular Medicine, Morsani College of Medicine, University of South Florida, Tampa, FL 33612, USA; 16 King Abdulazizi University, Faculty of Science, Department of Biological Science, Saudi Arabia; 17 International Institute of Nutritional Sciences & Food Safety Studies, School of Sport & Health Sciences, University of Central Lancashire, Preston, Lancashire, PR1 2HE, UK; 18 Maharaja Ranjit Singh Punjab Technical University Dabwali Road, Bathinda, Punjab, 151001, India; 19 School of Pharmaceutical Sciences, Shoolini University of Biotechnology & Management Sciences, Solan, 173229, India; 20 School of Pharmacy & Pharmaceutical Science, Ulster University, Coleraine, County Londonderry, Northern Ireland, BT52 1SA, UK

**Keywords:** nanomaterials, nanomedicine, therapeutics delivery, viral nanotechnology, viruses

## Abstract

The purpose of this review is to highlight recent scientific developments and provide an overview of virus self-assembly and viral particle dynamics. Viruses are organized supramolecular structures with distinct yet related features and functions. Plant viruses are extensively used in biotechnology, and virus-like particulate matter is generated by genetic modification. Both provide a material-based means for selective distribution and delivery of drug molecules. Through surface engineering of their capsids, virus-derived nanomaterials facilitate various potential applications for selective drug delivery. Viruses have significant implications in chemotherapy, gene transfer, vaccine production, immunotherapy and molecular imaging.

## Lay abstract

The purpose of this review is to highlight recent scientific developments and provide an overview of virus self-assembly and viral particle dynamics. Viruses are organized supramolecular structures with distinct yet related features and functions. Plant viruses are extensively used in biotechnology, and virus-like particulate matter is generated by genetic modification. Both provide a material-based means for selective distribution and delivery of drug molecules. Through surface engineering of their capsids, virus-derived nanomaterials facilitate various potential applications for selective drug delivery. Viruses have significant implications in chemotherapy, gene transfer, vaccine production, immunotherapy and molecular imaging. Here we performed a comprehensive database search to review findings in this area, demonstrating that viral nanostructures possess unique properties that make them ideal for applications in diagnostics, cell labeling, contrasting agents and drug delivery structures.

Viruses are obligate intracellular parasites. When a susceptible and permissive cell becomes infected with a particular virus, the cell cycle is redirected to produce additional viruses. All viral organisms have genetic material in either RNA or DNA format [1]. The genomic nucleic acid may be either single-stranded or double-stranded, circular or linear and either positive or negative in polarity. A virion is a complete component of the virus made up of nucleic acid and an outer protein layer that shields the nucleic acid inside. The amount of RNA or DNA present within a simple virus is sufficient to encode a few genes to generate viral proteins. As a result, the viral proteome does not surpass 100–200 proteins even in the most complex of viruses.

The origins of virology trace its beginnings to Dmitry Ivanovsky, over a century ago, on the persistence of new types of infectious matter in plant sap from Crimean tobacco displaying a standard mosaic patterned on infected leaves [2,3]. Although the plant sap was passed through membrane filters that retain microbes (mainly bacteria), the filtrate extract retrained the ability to procreate in plant cells once incubated with uninfected leaves [4]. Later, the virus of tobacco mosaic infection precipitated by Carl George Vinson (1927), and studies by Frederick Charles Bawden and Norman Pirie in 1936, showed that the virus comprises specific RNA and protein constituents [4,5]. Additional studies, sometime later, showed genetic material being retained is RNA molecules, so a definition of a virus was developed as the self-assembled particles of RNA and coat protein (CP). This particular virus is the tobacco mosaic virus (TMV), one of the most extensively studied and ubiquitous molecular pharmacological viruses of plants [6].

Viruses are considered mobile, genetically-engineered components, most likely intracellular and distinguished by a prolonged viral and host co-evolutionary relationship. An adaptive host cellular genome provides the diverse metabolic and biosynthetic functions of prokaryotic and eukaryotic species that are required during viral proliferation. The virion's primary function is to transfer DNA or RNA genomes through the environment to the host cell, where they are expressed (translated and transcribed) by the host cell. This gives rise to essential viral components, such as proteins and nucleic acids. The viral genome, often with accompanying basic proteins, is compressed into an asymmetric protein capsid. Along with the genome, nucleic acid–protein complexes form the nuclear capsid. Enveloped viruses have a fluid lipid bilayer formed by the transformed host cell membrane and an exterior surface of viral envelope glycoproteins encasing the nucleocapsid [7,8]. These viral capsids have attracted considerable interest as novel nanomaterials for drug delivery applications [9–12]. Therefore, this review summarizes the state-of-the-art and recent advances in this exciting field of viral nanotechnology, highlighting their unique properties, making them intriguing and naturally occurring nanomaterials.

## Viral structure & function

Viruses are inactive outside of the intended host cell, with simple types, such as polio and tobacco mosaic, existing in a crystallized form. Viruses do not contain the means to generate biochemical energy and depend entirely on the complex cellular pathways of prokaryotic or eukaryotic cells. The primary goal of the virus is to transfer its genome to the target cell through the host. The virus genome then encodes the capsid protein subunits and several specific viral proteins [13,14]. The genome encodes only for a few structural proteins (in addition to nonstructural regulatory proteins involved in virus replication) and is constrained in its size.

Capsids are composed of only one or a few structural protein types, assembled into single or double protein shells. Therefore, the continuous three-dimensional capsid structure must be constructed with several protein copies. The self-assembly of the virus capsids follows two simple patterns. The first is a helical symmetry, in which an extended helix is assembled. The second is icosahedral symmetry, in which protein subunits are assembled into a symmetrical shell that surrounds the center of the nucleic acid. Thus, the generated three-dimensional capsid is constructed from repetitive multi-protein subunit copies [15,16].

Certain classes of viruses possess additional structural features, known as an envelope. This typically originates from modified fragments of the host cell membrane. Viral envelopes are constructed from a lipid bilayer, which encloses a coat of membrane-associated proteins encoded by the virus. The outside of the bilayer is studded with mainly *trans* configured glycosylated membrane proteins coded for within the virus genome. Enveloped viruses frequently have pendant glycoprotein spikes or peplomers [17]. These surface lipid components closely reflect those found in the host plasma or intracellular membranes. Besides the envelope proteins defined by the virus itself, some host cell proteins are often retained by budding viruses as essential elements of the viral envelope [18]. The dynamic architectural configuration of larger viruses frequently consists of helical and isometric symmetries. Those much smaller viruses, such as hepatitis B, picornavirus or parvoviruses, tend to evade immunity more efficiently than the larger complex viruses, such as herpes virus or examples of retroviruses [19,20].

Viruses display asymmetry in their capsid configuration. For example, poxviruses are relatively large dsDNA viruses with complex morphology, seemingly missing any coordination in their brick- or oval-shaped capsids [21]. The vaccinia virus, which belongs to the poxvirus family, encodes more than 200 proteins. The arrangement of this virus was debated for some time because of its complex structure, large-scale asymmetrical arrangement, and sensitivity of the morphology to different environmental treatments. Vaccinia virions exist in three separate modes of infection, known as mature virions, enveloped virions and extracellular virions. Variation within the envelopes of each adds complexity to the structural features [22,23].

A mature virion is a brick-shaped entity, approximately 150–350 nm in dimension, comprising at least 75 different proteins. The intracellular types of a viral particle are known as mature virions. Other virions in cells, known as wrapped variants, consist of mature virions surrounded by membrane surfaces obtained from Golgi cisternae organelles. Wrapped virions end up leaving or exciting the cell by fusion with the cell membrane, generating an extracellular virion and leaving one of their envelopes behind in the process [24].

In addition to variation and irregularity in morphology, pleomorphic viruses embrace a wide variety of sizes, types and compositions. Owing to their inherent heterogeneity, the composition and structure of pleomorphic viruses can be computed by summing data from several similar viral particles, using x-ray crystallography and cryo-electron microscopy experiments, based on structural nanoparticle synthesis [25]. The emergence of electron tomography to image individual viral particles has revealed the architectural features of many significant human pathogens. Examples of structurally characterized viruses include representatives of retrovirus (HIV), orthomyxovirus (influenza), coronavirus (SARS-coronavirus) [20,26] and paramyxovirus (measles) [27–29]. Pleomorphism is prominent among enveloped viruses because the lipid envelope changes geometric patterns and proportions widely. However, protein-based self assembly may also contribute to pleomorphic capsids, which appear to form symmetric groupings. The retrovirus capsid protein (RPP) can be assembled into a hexamer and a pentamer, just like icosahedral viral capsid proteins [30].

New microbes thriving in extreme conditions, together with their related infective viruses, have been identified recently. Excitingly, there is a unique relationship between these organisms and their viral families. Examples found recently in Archaea have been gone unnoticed for some time. They are grouped into the fusiform, droplet- or bottle-shaped and linear viruses according to their standard class. There is no genetic sequence resemblance between viruses of different genome types, sizes and holding within each class. In environments populated by archaeal microbes, fusiform viruses are relatively abundant [31]. The virions with variable-sized tails protruding out from the spindle poles are spindle shaped. Acidianus two-tailed virus has an irregular appearance and can construct architectural new features until the host cell is eliminated. Isolated virions appear like ∼0.2 μm when propagated at temperatures marginally suboptimal for the host (75°C). However, if the temperature is high in the host cell's absence, these viruses expand from one pole to two filamentous threads of variable length. This is the only known example of an extracellular-assembled virus. However, there are undoubtedly others [32].

A conical center of a supercoiled nuclear protein structure is found in the enveloped ABV virion. The lower half of the bottle comprises a comb of small filaments, but then the host binding is visible on the other side. The SNDV virion design is poorly understood. Simple archaeal viruses will eventually form vertical rods or flexible filaments (Rudiviridae) (Lipothrixviridae). In terms of structure, rudivirus is surprisingly simple, with no envelope and just a few proteins organized in variable-sized particles, usually linked to genomic molecules. Lipothrixviruses are covered with frameworks in various types (spider wings, pincers, bottom) and are capped at the ends of their filamentous nucleocapsid [33,34]. It has been reported that liposomal nanomaterials are generated from cholesterol and phospholipids isolated from archaeal lipids of *Sulfolobus islandicus* rod-shaped virus. The virus naturally propagates in a harsh environment and presents an ideal nanocarrier for compounds, such as peptides and proteins, through the oral delivery route. The formulated liposomes are stable in the presence of bile salts and have been shown to preserve a model payload (calcein) [35].

### Plant virus structure

Plant viruses are either DNA or RNA viruses, consisting of one protein coat and one nucleic acid type. About 75% of genomes of plant viruses consist of single-stranded RNA (ssRNA). Although more than 65% of plant viruses have a positive-sense of ssRNA, 10% have a negative sense. Plant viruses with a genome of negative ssRNA must be transformed to positive ssRNA before translation is initiated [36,37]. The positive sense double-stranded RNA of 5% of identified plant viruses is translated automatically. An inverse transcriptase enzyme is required to transition from RNA to DNA in roughly 3% of plant viruses. Some three-quarters of identified plant viruses are ssDNA, but a few have the double-stranded genome of DNA (dsDNA), such as the Caulimoviridae family [38,39]. By way of comparison, almost a quarter of animal viruses have such a dsDNA genome [40].

**Figure 1.**
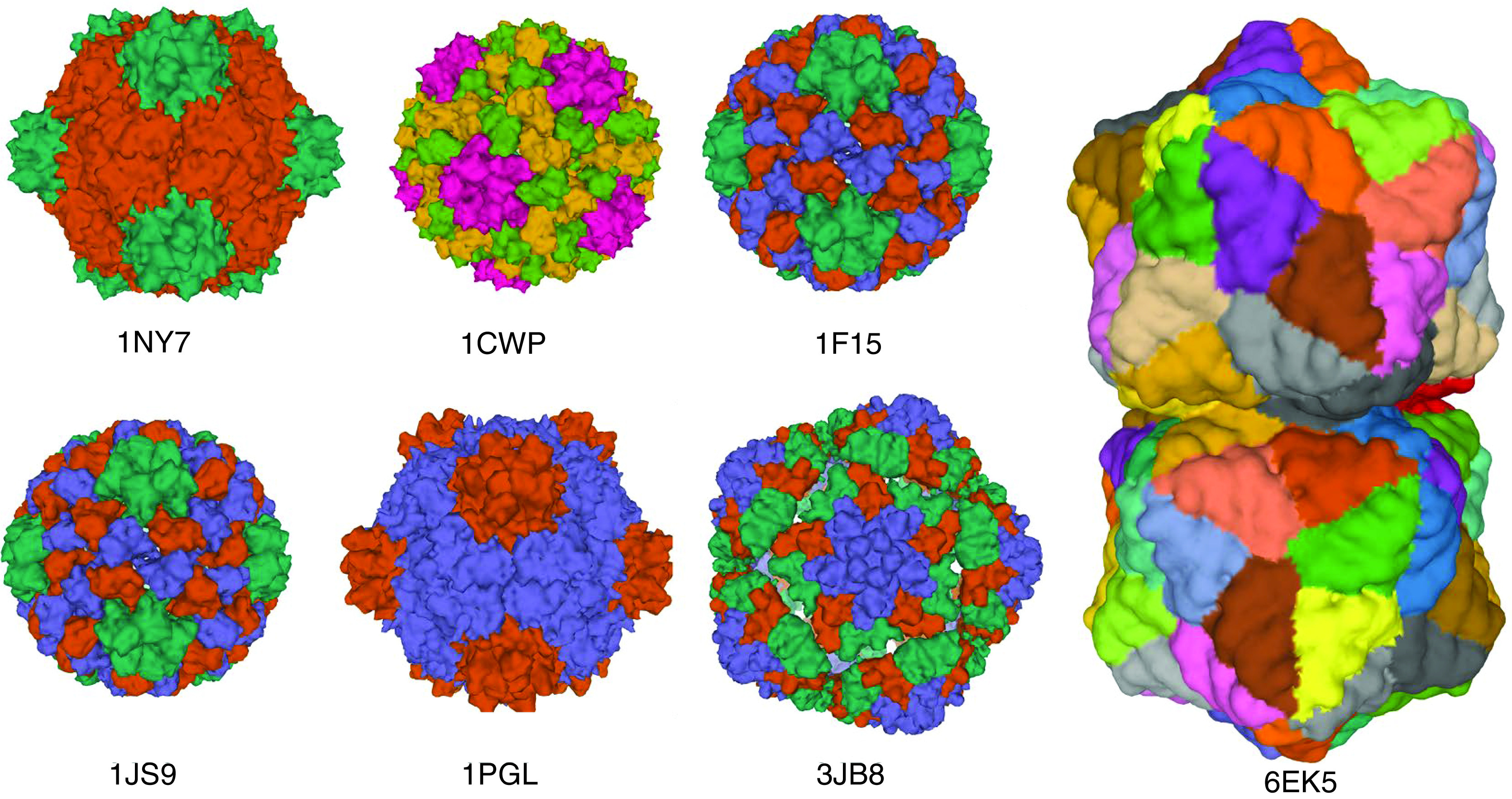
Schematic illustration of some examples of plant viruses with their corresponding Protein Data Bank entry code. Cowpea mosaic virus (1NY7), cowpea chlorotic mottle virus (1CWP), cucumber mosaic virus (1F15), brome mosaic virus (1JS9), bean pod mottle virus (1PGL), maize chlorotic mottle virus (3JB8), and plant Gemini virus (6EK5). Images were generated from the Protein Databank with the reference accession numbers with PyMol software.

The viral arrangement comprises genome coat proteins capable of spontaneous assembly into the viral particles of specific configurations, as shown in Figure 1. More than half of identified plant viruses have a rod shape. While the length of viral particles usually is genome size-dependent, it typically ranges from 300 to 500 nm, with a diameter of 15–20 nm. The protein subunits are packed to form a tubular-like construct, with a hollow cavity in the genome center [41,42]. The single most abundant construct among plant viruses after the rod-shaped isometric particles is the icosahedral particle with a diameter of 17–75 nm. The fundamental structure consists of 60 subunits for plant viruses with a single protein coat only. The number of subunits is clearly an integer value [43–45]. Simultaneously, these two subunits construct an icosahedral particle in plant viruses with two types of coat proteins [46]. The Geminiviridae virus family represents better-studied examples of the latter case. They have unique dual particle geometries, entailing two isometric particles in combination. In addition to their coat proteins, a few plant viruses have a lipid envelope. The Tospoviridae family is a typical example of enveloped plant virus, leading to substantial economic losses in terms of agricultural production [47,48]**.**

## Production of virions & virus-like particles

**Figure 2.**
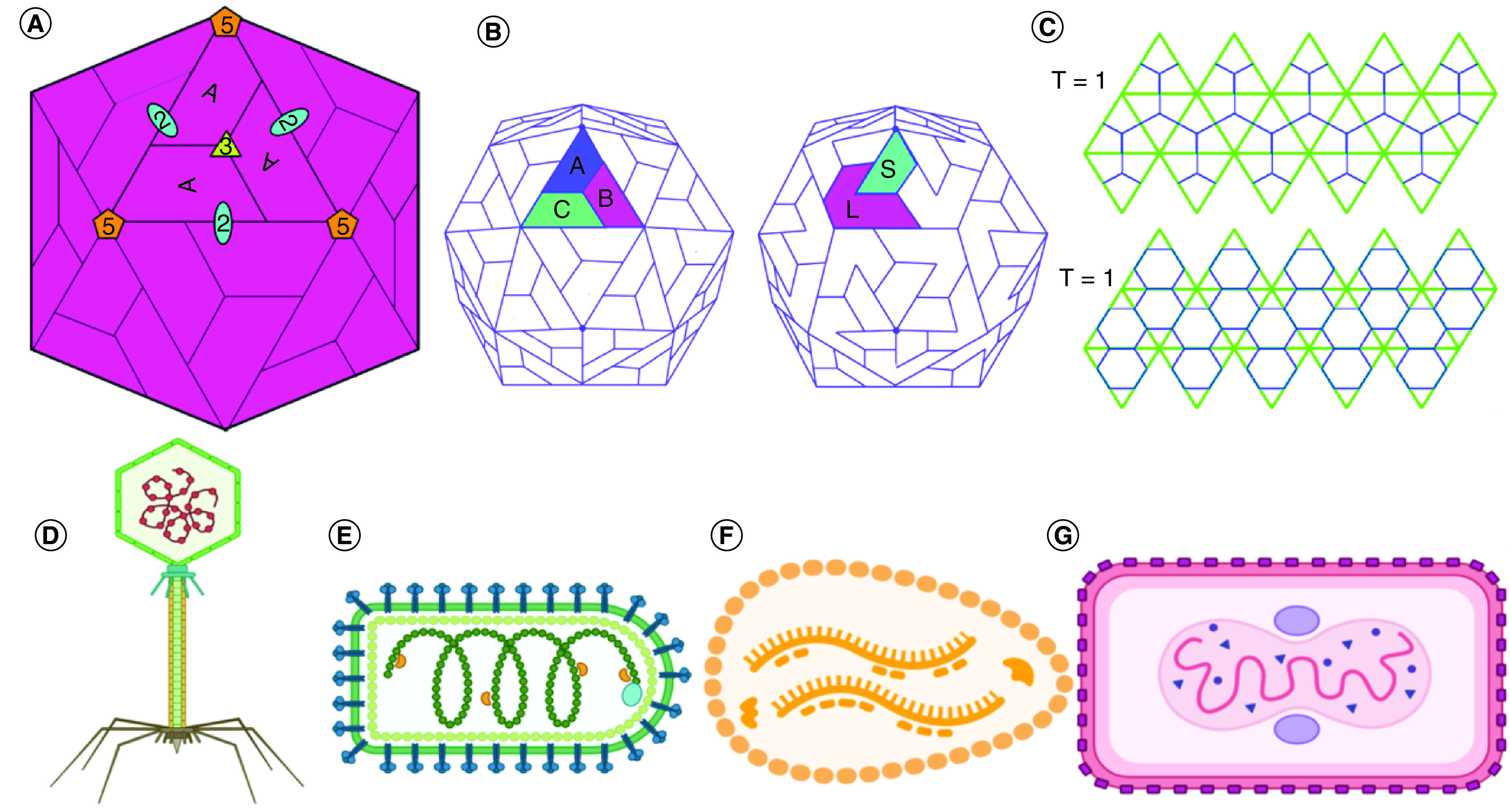
Different icosahedron viral nanoparticles. **(A)** Schematic drawings of icosahedron particles showing the twofold, threefold and fivefold asymmetric units made of one type of proteins and multiple axis. **(B)** Schematic drawings of icosahedron viral symmetry. B is T = 3. **(C)** Flat view of virus symmetry and the T number based on Caspar–Klug theory for the viral assembly. 60 identical subunits self-assembled on the 20 triangles creating the face of the icosahedron. The generated icosahedron geometries exhibit asymmetry around the fivefold, twofold and the threefold T = 1 and T = 7 icosahedron capsid are showing the triangular faces with the asymmetrical unit outlined. **(D-G)** An array of complex viruses' examples showing their different geometrical arrangements and their exterior shape.

An outstanding demonstration of effective use of genetic capacity and controlled self-assembly is the mounting of viruses from a limited group of subunits, in which the final structure of the substance is programmed in the sequence of nucleotides. A specific arrangement of protein molecules determines the capsid size, orientation, morphology and shape of the viral capsids, as shown in Figure 2A. The assembly method also often relies on the interaction with the capsid cargo of nucleic acid or other structural proteins that gives the capsid protein considerable potential [49]. In this respect, the theoretical and *in silico* activities are focused on clarifying the viral assembly processes in the presence of a cargo molecule. Numerous studies describe findings that approached the assembly process utilising models consisting of specified natural or synthetic cargoes.

Dragnea and co-workers use the *in vitro* reversible brome mosaic virus (BMV) assembly to assess the scale of capsid assembly capacity by comparatively unspecific electrostatic interactions of the coat proteins with the nucleic acid payload. BMV virus-like particles (VLPs), each retained the icosahedral arrangement, with the triangulation (T-numbers) depending on payload size, could be generated using gold nanoparticles of specified sizes, decorated with glycol with a negatively charged terminus as replacements for the natural nucleic acid load. This packaging versatility was not exclusive to the cargo of gold NPs, and functional iron oxide particles could be equally encapsulated [50,51]. Although the BMV's normal morphological stability backs these experiments, the inference is that cargo interactions and coat modulate coat-coat interactions and determine the resulting structure that illustrates the exciting potential to broaden the structural functionality of VLPs [52]. By organizing symmetrical interactions, cages are formed. The structural equation model for icosahedrons, the spherically symmetrical viruses, is reflected in a single replicated subunit with sixty subunits of five, three and two rotational symmetry straightforward series of interactions required to render the enclosed container cage model. Each VLP family is characterized by a particular collection of symmetric interactions between subunits. In this respect, the efforts to build new cages by fusing protein domains with an established or planned quaternary structure have been made successfully. Yeates *et al. *have designed numerous protein cage systems by combining the protein domain with a known propensity to follow established quaternary structures (two-, three- or fourfold interactions). These efforts created cages with specified octahedral or tetrahedral symmetry by integrating protein prediction simulations and Rosetta design tools with genetic structures [53]. They successfully engineered cages of two distinct subunits of proteins to extend their approach's available complexity. While the *de novo* design of increasingly complex capsids is currently restricted to relatively small cage architectures, it is possible to understand the required interactions in subunits [54] accurately.

Plant viruses are fascinating because they are not pathogenic agents of human and animal subjects. Plant virus composition is relatively simple and consists of a single coat protein. Most have a very high aggregation amount on their host and are not enveloped. The theoretical use of VLPs derived from plant viruses as a novel protein scaffold has been investigated in recent years to produce novel epitopes. Foreign immunogenic peptides can be incorporated to plant virus coat protein either genetically or chemically. The VLPs were screened based on multiple plant viruses, including Cowpea mosaic (CPMV) virus, ALFA mosaic virus, potato virus C, tomato bushy stunt virus, plum pox virus, chlorotic cowpea mottle virus (CCMV). VLPS assembly emerges under some parameters, such as pH, ionic strengths and viral genomic RNA interaction [9].

Production of plant virus VLPs was established in different microbial expression systems, including *E coli*, *Pseudomonas fluorescens*, *Pichia pastoris* and *Saccharomyces cerevisiae*. It is recognized that the expression mechanisms in prokaryotic and eukaryotic organisms have benefits and disadvantages [54]. In fact, in prokaryotic systems, production is usually economical and can be completed quickly. The inability to change the resulting structures after translation and the existence of endotoxins are the most exciting concerns when they are used. Besides, because large amounts of protein are sometimes manufactured in an insoluble state, different denaturation processes and refolding may be needed as in the CCMV case. The amount of protein produced is typically less than in *E. coli* regarding the yeast expression method. Soluble proteins are nevertheless obtained without further solubilization [55,56].

### *In vitro* viral assembly

Coat components of many plant viruses, including the well-studied TMV, create VLPs *in vitro* through self-assembly. TMV assembly is initiated by the interaction of a capsid protein oligomer with an origin-of-assembly region (OAS) located 900 nucleotides from the 3′ terminus of the virion RNA. This assembly can be achieved *in vitro*. These attempts have been made to assemble rod-shaped particles with modified cover protein that give the subsequent rod-like particles desirable properties. Eiben *et al.* noted that it was possible to construct mutant rods by combining *E. coli*-synthesized mutant coat protein [57]. Wild-type coat protein *E. coli* derived in the assembling reaction from a plant TMV infection. The flexural rod and icosahedral plant viruses, which permit various foreign species' encapsulation, have been utilized in a related *in vitro* configuration, and different plant viruses were explored as nanocarriers of useful cargo. Filamentary viruses may be changed to create new structures, modifying biophysical features of the resulting nanoparticles and *in vitro* assemblies to create particles with wild-type geometries. The most significant example is the development of different nanospheres from the TMV rod virus. Plant viruses have a long record of being used as carriers for a range of nanotechnology and tissue engineering, which we divided into three various categories: medicinal provision, bioimaging and metallization [58]. Transportation of cargoes is a vehicle for delivering target molecules utilizing the internal viral cavity; however, the incorporation of specified cargo in the virus is nontrivial. The infusion technique aims to disperse a load of interest onto preformed viral particles, while the method for caging triggers particulate forming from around content load. It also aims to encourage plant viral particles' packaging with external loads [59,60]. The genetic engineering of TMV-Lys at position 158 has been utilised as a scaffold for the bioconjugation of sulfo-Cyanine5 fluorescent tag for fluorescent imaging that laid the foundation for developing rod-shaped viruses as drug carriers scaffolds [61].

### *In vivo* viral assembly

VLPs are assemblies with automatic and specific self-assembly mechanisms. They lack a viral genome and are generated in heterologous expression systems (e.g., *E. coli*), yeast strains, plants, mammalian cells and insect cells. They reflect a transition to noninfectivity. VLPs resemble wild-type viral particles, both architecturally and morphologically, sharing joint tropism, absorption and intracellular distribution in cells [62]. In some instances, pH-induced swelling and alkaline hydrolysis of released nucleic acids can also lead to the production of VLPs from wild-type viruses.

Consequently, preserved viral particles can be broken up and reassembled into VLPs under appropriate conditions, such as temperature, protein concentration, pH, solution ionic strength and macromolecular crowding components throughout the specific subunits of the viral coat protein [63]. VLPs are safer than pathogenic viruses in vaccine development. As a result, VLPs have no limitation of replicating or resorting to virulent phases as conventional vaccines would do. VLPs reflects pathogen-associated molecular patterns due to their capability to conform to multimeric structures like wild-type viruses in terms of immunity. The target host cells may discriminate against VLPs and trigger a receptive immune response [63]. Although produced within a range of heterologous expression systems, the bacterial expression does not typically have post-translational processing and does not support glycosylation of target proteins, which may be essential to produce a proper immune response following translation [64,65]. Mammalian cells are often used as a mechanism of expression with a relatively low disadvantage relative to bacteria or systems of expression of baculoviruses (0.018–10 μg per ml) [66].

## Physiochemical characteristics of viruses

Viruses take different shapes and forms. These define their ability to evade an immune response, invade target cells and deliver their payload to the host cell. Viruses are classified morphologically into two types, namely spherical or rod-like. More generally, viruses are categorised based on their morphology, chemical composition, structure and form of replication. Presently, viruses infecting humans are classified into 21 families and constitute a small portion of the continuum of numerous viruses whose host range spans from vertebrates to protozoa and from plants and fungi to bacteria [3,67].

### Morphology of viruses

When viewed using electron microscopy, helical viruses appear as rods. The rod can be rigid or flexible. The tobacco mosaic virus (TMV) is the most studied example of a simple helical virus. TMV is 18 nm in diameter and 300 nm in length with a rigid rod configuration. It comprises 2130 subunits consisting of a particular 17.5 kDa capsid protein. Every protein subunit has the nearest six neighbors in the right-hand helix. Except for these subunits at the helix ends, every capsid protein subunit occupies an equal location in the resultant network [68]. Most viruses have helical symmetry and sometimes possess only one or very few proteins. Since the particle's length is not fixed and standard and RNAs or DNAs of various sizes can easily be modified, the lattice's prominence can be attributed to its length [69,70]. The roles of morphology and shape of viruses in their biodistribution and duration of the bloodstream circulation were evaluated. For example, it was shown that the rod-shaped structures have more circulatory time in the bloodstream in mice and are eliminated less rapidly from tissues than spherical constructs, implying that the viral shape plays an essential role in biodistribution, mainly as a drug carrier [71]. For host evasion, the size of viral particles is also a crucial element as a carrier. Macrophages, dendritic cells, monocytes and other cells necessary for virus clearance efficiently engulf particles, particularly particles in the micrometer scale, in terms of size. In addition, virus diameter influences the path to cellular uptake. Macrophages take up large viruses such as mimivirus (∼760 nm in diameter), while smaller viruses are typically internalized in cells through clathrin-mediated endocytosis [72]. Viral particles' surface charge and hydrophobicity are also essential for immune evasion. Cells are more susceptible to hydrophobic particles than non-ionic, hydrophilic ones. Positively charged particles have more extended circulatory periods than negatively charged particles, which shows that surface charges influence viral circulation time, depending on the environment surrounding the surface charge of the virions. The surface charge of the viral protein is variable even from a negative to a positive charge, depending on the isoelectric point (pI) of viral protein regulated by the environmental pH [73].

To generate an icosahedron, an isometric structure is assembled from 12 pentagonal vertices and 20 triangular faces. Any icosahedron seems to have an exact set of symmetrical elements: six five-fold axes on each of the 12 vertices, ten triple axes on the 20-triangle end and 15 double axes [74] corners, as shown in Figure 2B. A structure containing 60 equivalent subunits is the simplest icosahedral structure see Figure 2C. Caspar and Klug [75] noticed that all capsid subunits would not have the same microenvironments if more than 60 subunits combine to form a closed shell, as many spherical viruses have demonstrated.

**Figure 3.**
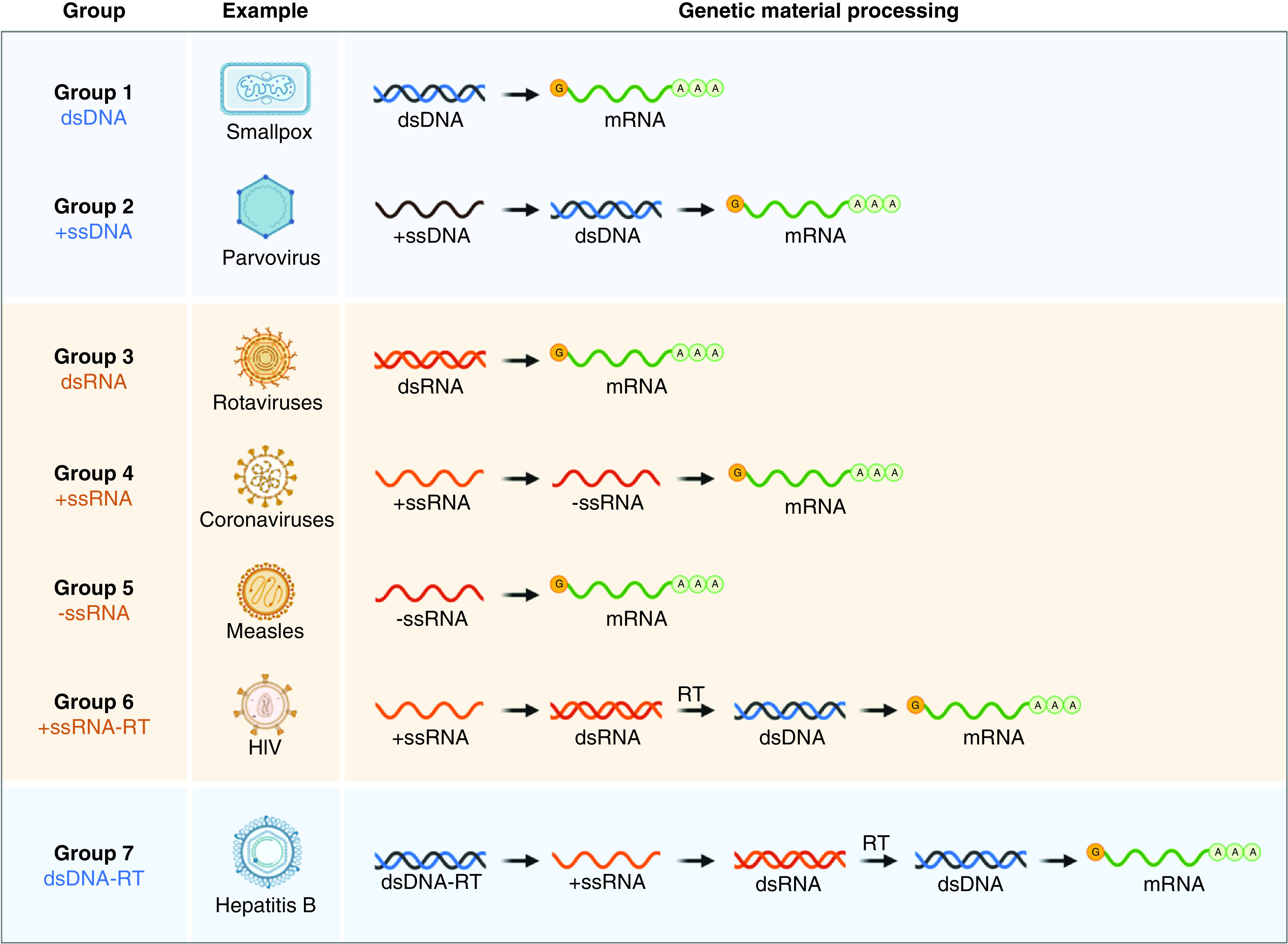
The Baltimore classification system divides viruses into classes depending on their gene type and replication strategy. A virus must produce mRNAs from its genome to reproduce, and there are seven ways to do so or seven types of viruses. Group 1 viruses can transcribe mRNA directly from their ds DNA genomes, while Group 5 viruses must transcribe their negative sense ss RNA genomes using an RNA-dependent RNA polymerase. All Group 6 and Group 7 viruses utilize reverse transcriptase enzymes in the replication processes of their ssRNA and dsDNA genomes. ds: Double-stranded; RT: Reverse transcriptase; ss: Single-stranded. Created from reference [82] using Biorender.

The phage tail fibers can also occur in complex viruses but are now paired with several diverse capsid proteins needed for a proper virion assembly. These proteins could be treated as disconnected molecular switches to attenuate the diversity of associations necessary to assemble the complex capsid and ensure its stability as shown in Figure 3. One situation concerning the intricate capsid arrangement is (i) biochemically distinct hexameric or pentameric capsomers; (ii) a phage tail fiber platform; and (iii) conjugation of polypeptide chains [76].

### Viral chemical composition

Nucleic acid (genome) and protein are the essential components of infectious virions. In contrast, in their glycoprotein peplomers, all enveloped viruses contain lipid and carbohydrate. Lipids in other parts of the virus are associated with the largest and most complex viruses (poxvirus using ranavirus and African swine fever virus) [77].

Every unique virus contains only one nucleic acid type. However, it may be DNA or RNA since the only occasion in which RNA is the only repository of genetic material is the RNA virus. They produce only a single copy of the chromosome, except for the diploid retrovirus genomes. Double-strand (ds) or single-strand DNA or RNA may be single- or double-strand as well [78]. The nucleic acid in viruses can only replicate in the target host cells in both DNA and RNA viruses; that is, the entire viral replication cycle can be triggered upon entering the cell to generate viral progeny. In this scenario, mRNA in a nuclear, cellular transcriptase, and genomic RNA acts like mRNA, which is the viral DNA transcribed in the nucleus [79]. In the virion, the capsid proteins are clustered together to make capsomers visible in the electron's micrographs. Each capsomer has one to six polypeptide molecules, typically the same form (homopolymers) but often different (heteropolymers) [80].

### The Baltimore classification of viruses

While certain viruses are divided into specific families based on several physical and biological factors, they may also be classified based on the form of genome included in the virion. David Baltimore, a virologist, developed an alternate classification system about 30 years ago that considers the existence of the viral nucleic acid. Understanding how viral genomes are distributed has been one of the most notable developments in virology over the last 30 years. Cellular genes are encoded in dsDNA, from which mRNAs are synthesized to direct protein synthesis. To generate proteins, all viruses must guide the synthesis of mRNA. Since no viral genome encodes a complete system for protein biosynthesis, all viral protein synthesis is entirely dependent on the cell's translational machinery. Baltimore developed his virus classification scheme focused on the function of viral mRNAs in programming viral protein synthesis and the central position of the translational machinery [81]. The Baltimore scheme originally included six classes of the viral genome, as seen in the figure. Following that, the DNA genome of hepadnaviruses (e.g., hepatitis B virus) was discovered. The scheme's rules are a precice illustration of studying the knowledge flow in viruses of various genome configurations. Knowing only the viral genome structure reveals the essential steps that would occur to generate mRNA.

### Generic viral life cycle

**Figure 4.**
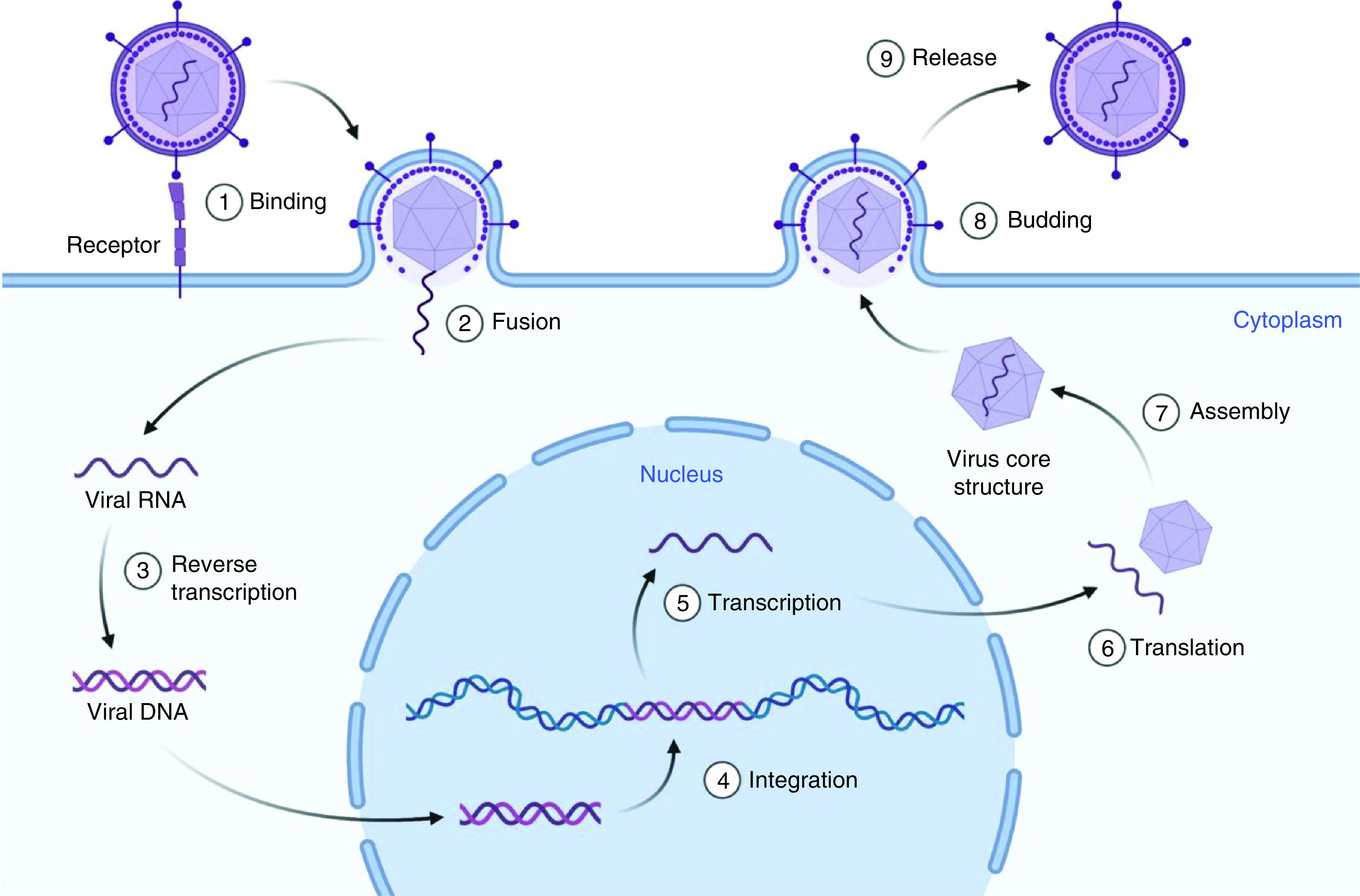
Generic life cycle of a virus initiated with viral attachment to host cell followed by viral entry, replication, maturation and release. Created using Biorender.

As obligate intracellular pathogens, viruses depend on host cells for replication and metabolic processes. Virus life cycles vary widely depending on the species and form of the virus, but they do adopt the same basic steps for viral reproduction. Attachment, entry, uncoating, replication, maturation and release are the main phases of the viral life cycle. The viral genome must be passed from a virus particle into the cytoplasm of a host cell until a virus can invade it. Viruses enter eukaryotic after being attached to cell surface receptors. Few viruses invade cells by specifically fusing with or invading the cell membrane, but the majority of viruses enter by endocytosis (coronaviruses can enter both ways) (Figure 4) [83]. This endocytosis is most commonly accomplished through clathrin-coated pits, from which cargo is shuttled to progressively acidic organelles, ranging from early endosomes to late endosomes to lysosomes [84]. Coronaviruses, for example, may fuse at various stages of endocytosis: MERS coronavirus fuses with early endosomes, while SARS coronavirus fuses with late endosomes. Viruses that need an even lower pH would not fuse before they reach the lysosome. These endocytic and lysosomal vesicles have a low pH and abundant proteases, which may cause a virus's conformation to shift, resulting in uncoating, fusion or penetration into the cytoplasm. Even viruses that do not need a low pH use endocytosis as a quick way to cross the plasma membrane and transit across the cytoplasm to their replication sites [85].

Once within a cell, a virus starts the process of uncoating and reproduction. Viruses, as intracellular obligate pathogens, must regulate cellular proteins and organelles in order to replicate. Replication centers coordinate the cellular components necessary for viral gene expression and replication [86]. Some viruses multiply in the cytosol, while others, such as herpesviruses, adenoviruses and influenza viruses, must navigate their way to the nucleus [87]. Viruses employ various methods to extract the viral genome from the virion, the complete, infective type of a virus outside of a host cell (Figure 4). Certain viruses, such as rhinoviruses, extend to form endosome pores from which the viral genome may escape. Influenza and other viruses cause the virion envelope to fuse with the endosomal membrane, allowing the viral genome to be released.

## Viruses as drug carriers

Conventional drug carriers have been licensed and developed over the past three decades based on lipid-based, polymeric, organic and inorganic and protein-based nanomaterials [20,88]. To develop clinically viable drug carrier systems, such systems must be biodegradable, biocompatible and efficient in therapeutics delivery. Although there is no ideal therapeutic delivery developed yet, protein-based nanomaterials have been developed as potentially viable clinical therapeutics agents. Some of the proposed materials have already been evaluated in clinical settings as imaging agents while evaluating their cytotoxicity profile [65,89,90].

**Figure 5.**
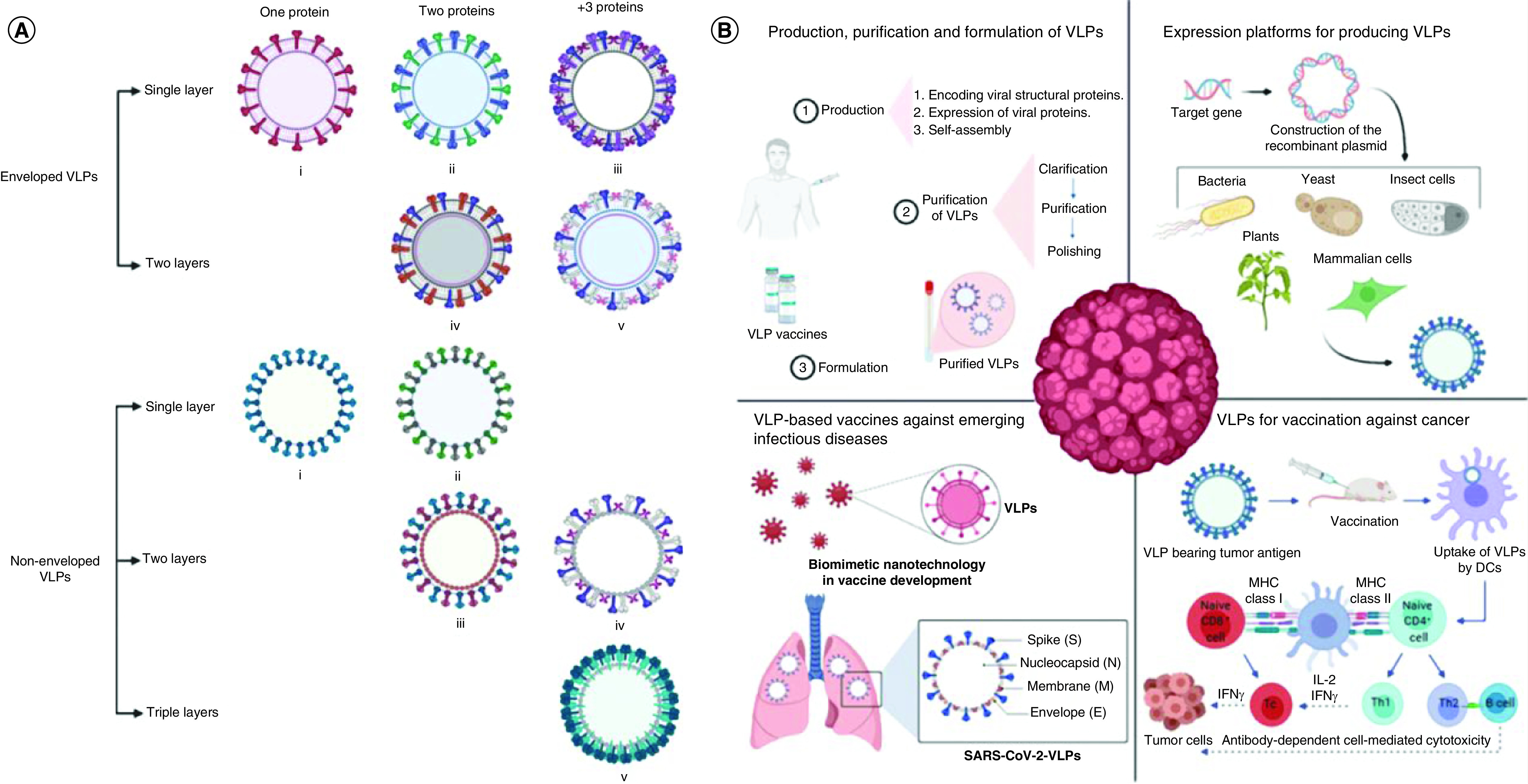
Schematic illustration of various virus-like particles. **(A)** Classification of various virus-like particles (VLPs) structures. **(B)** Recent advancements in the production and manufacturing of VLPs include the investigation of various forms of expression mechanisms for their growth, as well as their applications as vaccines in the prevention of infectious diseases and cancers as a consequence of their association with, and process of activation of, the humoral and cellular immune systems. DC: Dendritic cell; MHC: Major histocompatibility complex; VLPs: Virus-like particles. Reproduced with permission from [91].

In specific, these attractive properties are correlated with viruses, including noninfective VLPs (Figure 5) [91]. They will self-assemble into specified geometry configurations, display a role of spatial stability to permit protein species to work and provide responsive amino acid sidechains to be used to conjugate synthetic or less desirable species (Figure 5A) [92]. VLPs are virus-derived structures composed of one or more distinct molecules that can self-assemble, mimicking the shape and scale of a virus particle but missing the genetic material to invade the host cell. The viral structural proteins can be expressed and self-organized in various living- or cell-free expression environments, after which the viral architectures can be assembled and rebuilt. VLPs are gaining interest in the field of preventive medicine, and to date, a wide variety of VLP-based candidate vaccines for immunization against different infectious agents have been created, the most recent of which is the vaccine against SARS-CoV-2, the effectiveness of which is currently being assessed (Figure 5B). VLPs are highly immunogenic, eliciting antibody- and cell-mediated immune responses by separate mechanisms than traditional inactivated virus vaccines. However, several issues with this surface display device must be resolved in the future – VLPs, known as subunit vaccines, are further subdivided into enveloped and nonenveloped subtypes covered in this review paper. VLPs have recently got quite a bit of recognition with their promising applications in selective drug delivery and gene therapy [93]. The creation of more efficient and selective types of VLPs by altering the particle surface to be inserted into particular cells or tissues or extend their half-life in the host is likely to enhance their usage in the future.

The use of plant viruses decreases much of the potential consequences via bioactive molecules for medical nanotechnologies and results in decreased potential damage due to noninfective VLPs. Those reduced threats make it easier to treat, hold and handle viral nanoparticles and make plant virus-based particles especially attractive frameworks for many nanobiotechnological sciences implementations [94].

**Figure 6.**
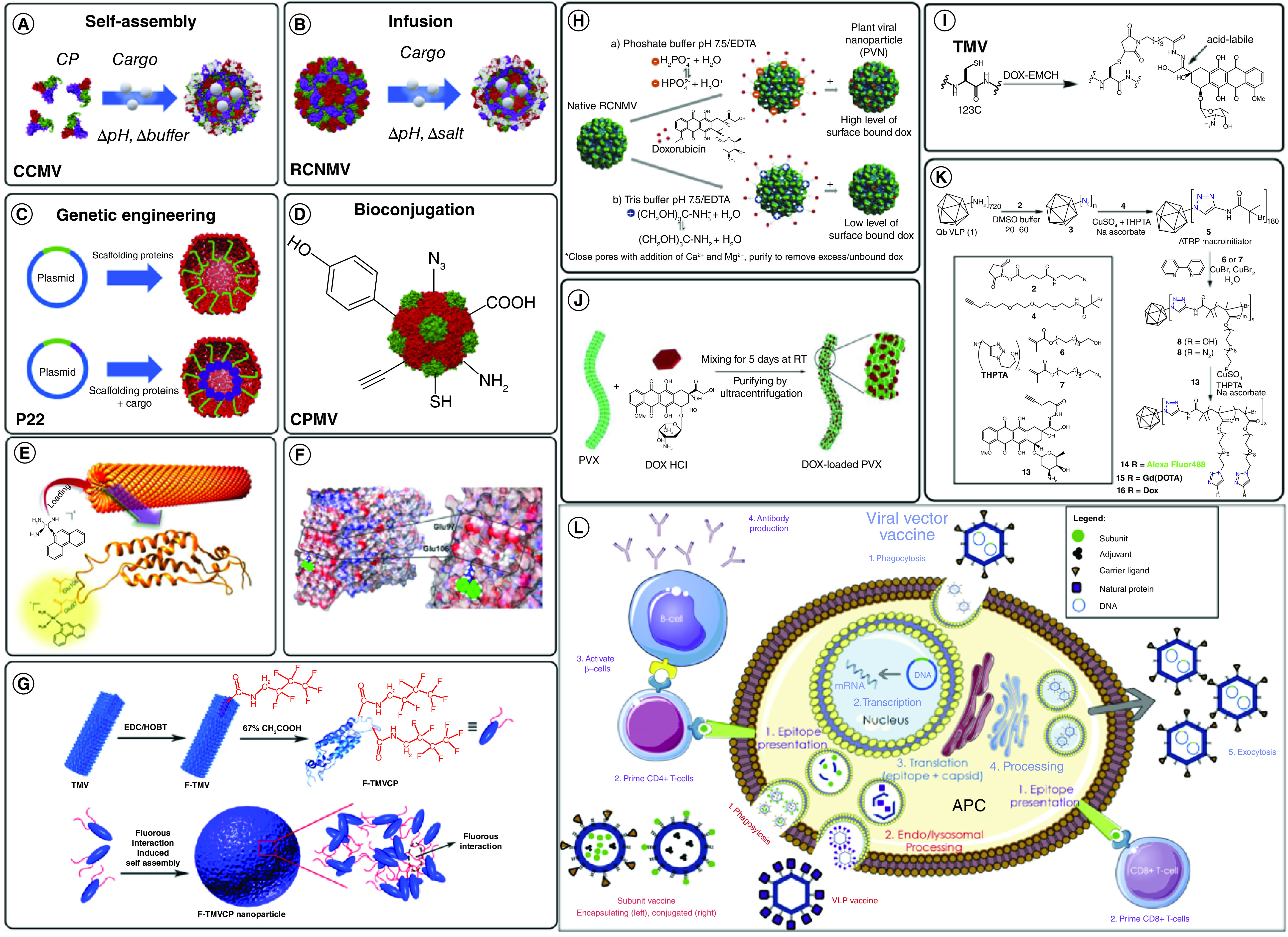
Strategies for carrying cargo with virus-like particles. **(A)** Self-assembly around cargo by varying pH and buffer conditions with congenital cytomegalovirus. **(B)** Infusion of cargo inside red clover necrotic mosaic virus due to changes in pH and salt concentrations. **(C)** Genetic engineering strategies that use genetically conjugated scaffolding proteins to encapsulate drugs inside P22. **(D)** Bioconjugation into cowpea mosaic virus using exterior surface-exposed residues loading platinum-based drugs such as phenPt and cisplatin into tobacco mosaic virus (TMV) via various mechanisms. **(E)** Graphical abstract from Lippard *et al.* illustrating the loading diagram for phenPt loading onto wild-type TMV particles. **(F)** PhenPt docking into TMV Glu97 and Glu106 residues discovered using matrix-assisted laser desorption/ionization – mass spectrometry and nuclear magnetic resonance spectroscopy. **(G)** Assembly of tobacco mosaic virus spherical nanoparticles through a fluorous ponytail interaction (F-TMVCP). Doxorubicin (DOX) incorporation into various virus-like particles (VLPs) via infusion, bioconjugation, adsorption and polymerization chemistries. **(H)** Surface attachment of DOX onto red clover necrotic mosaic virus VLPs in various buffers to investigate the binding release properties of DOX from the nanoparticles. **(I)** Bioconjugation of DOX-N-maleimidocaproic acid hydrazides onto TMV disk VLPs through cysteine residue maleimide linkages. **(J)** Adsorption of DOX onto the exterior surface of potato virus X. **(K)** Copper-catalyzed azide-alkyne cycloaddition of DOX into OEGMA-N3-polymerised Q. **(L)** Mechanisms of action of various viral vaccine candidates. CCMV: Congenital cytomegalovirus; CPMV: Cowpea mosaic virus: DOX: Doxorubicin; PVN: Plant viral nanoparticle; PVX: Potato virus X; RCNMV: Red clover necrotic mosaic virus; TMV: Tobacco mosaic virus. Reproduced with permission from [100].

Viral nanoparticles (VNPs) are nanomaterials generated spontaneously by plant viruses, bacteriophages and mammalian viruses. VNPs and their genome-free equivalents, VLPs, are rapidly being used and advanced in nanomedicine. VLPs may encapsulate a range of active ingredients and be genetically or chemically conjugated to targeting ligands (see Figure 6 for different techniques used for the virus as drug carriers) [60,95–99]. Scalable fermentation or molecular farming was used to create VLPs, and the products used are biocompatible and biodegradable. These properties have aided in developing a wide range of applications, including cancer drugs, immunotherapies, vaccines, antimicrobial medications, cardiovascular medicine, gene therapies, imaging and theranostics. VLPs are being used as medication delivery devices, and sufficient research must be done continuously to get these treatments to the clinic.

Besides serving as construction blocks for materials, VLPs will naturally also serve as containers for the limited nanoparticles' synthesis composed of other materials. Ferritin is an octahedral small spherical VLPs. Ferritin is almost omnipresent, catalyzing iron oxidation and storage that can be cell toxic [101]. Only the protein subunits that shape the cage structure include enzymatic ferroxidase sites and give high-charge nucleation density sites to produce the resulting iron oxide nanoparticle. Ferritin exterior cages are inactive nucleation and pores in the cage cause ions to pass through the shell and enter inside. A simple protein subunit activates all these specifications to organize these functions when assembled into a closed shell cage. *In vitro* models of the mineralization phase ferritin have demonstrated promiscuity against numerous abnormal mineral nanomaterials that are reactive. Ferritin facilitates development under biological conditions of a kinetically trapped iron oxyhydroxide polymorph (ferrihydrite) [102]. Approaching to access iron oxide alternative polymorphs through changes in the reaction conditions using empty ferritin (apo-ferritin) was created. These ideas have been used to successfully nucleate and expand a wide range of other non-natural minerals in ferritin cages.

The internal space for the VLPs allows for the templated and limited summary of polymer networks in addition to inorganic nanoparticles. In the shape of a viral genome, virus capsids have developed to contain an expanded biopolymer. Since the release of large polymers is restricted to small molecules, the cages remain widely permitted. This function permits polymerization situated inside the VLPs by specific monomer production at the initiation sites inside the cage [103,104]. The genetical origin of VLPs makes it possible to selectively create several polymer initiation sites within and outside the cage utilizing ready site-directed mutagenesis. Some VLPs are exceptionally tolerant of organic solvents and extreme conditions of the reaction. As such, there are various available methods for marking the cage and polymerizing the inside. The VLPs have modifiable surfaces both within and outside of the particle, as previously discussed. A joint of freight can be carried out in a synthesis, genetics or mixture of the two surfaces. Routes to cargo bioconjugation can use external amino acid side chains, amino acids added and non-natural amino acids or peptide termini implemented. Some VLPs tolerate organic solvents, elevated temperature and a broad range of pHs, such that they are compliant with several different reaction conditions [105,106].

The payload of the freight is increased as a soluble cargo is loaded into a nanoparticulate. If a drug candidate is needed to obtain the desired effect of 100 molecules per cell, then the cell must be penetrated individually by 100 different molecules. However, if a nano part with 100 molecules is prepared, each nano part hitting the cell theoretically provides the payload and the desired result. Moreover, many surfaces and special chemical groups provided by several nanoparticles permit both a collection of cargo molecules and a set of molecules aimed at the aggregation of particles at a particular location [107,108].

Viruses already filled with nucleic acid have been repurposed with molecules native to the genome. The loading and distribution by combining encapsulated nucleic acid in CPMV of several fluorescent dyes and therapeutic proflavine have been achieved. Related methods were used for the transmission of doxorubicin in the cucumber mosaic virus [109]. toward improving the MRI comparison, lanthanides were used as a natural affinity for loads of nucleic acids Qβ and CPMV with Gd, and the capsids were loaded additionally with Gd in Qβ via azide-alkyne Click Gd-chelate combination to capsid sites. This performance is strongly determined by the bioconjugation reactions for marking and resolving the structural capsid details available for design [110,111]. While empirically determinable marking positions, prior observations into the position of locations inside the protein structure vastly boost the capacity to mark the capsid in such a manner that the cargo is located inside or outside. CPMV was one of the first VLPS originating from viruses to be used in bioconjugation. CPMV provides an easily generated VLPS system with a large scale of structural characteristics with a high resolution and stability to a broad spectrum of disturbances. CPMV initial experiments used natural residues of lysine to bind poly-ethylene glycol (PEG), biotin, or fluorescent molecules to guide, track and assemble higher cognitive viral material throughout the particle's immunogenicity [112,113].

In order to enhance pharmaceutical efficiency and suppress systemic toxicity, cathepsin B-cleavable DFK peptides were deployed on the p8 coat proteins of the filamentous phage, which were used to attach doxorubicin with the high copy number (∼3500) via carbodiimide conjugation chemistry to establish a linker that is highly serum stable and sensitive to enzyme hydrolysis by cysteine protease (cathepsin-B; present in the lysosomes of target cells). Although the direct conjugation of the drug with the coat proteins generated more molecules per phase (∼10,000), the drug release mechanism developed dramatically enhanced carrier capacity following the release of the pharmaceutical drug into the target cells [114].

Studies using bioconjugation strategies have also demonstrated the various utility of bacteriophages MS2 in cargo and cellular supplies [115]. Peabody and colleagues combined tempered VLPs assembly with co-labeling of capsid proteins to target peptides, quantum dots, siRNA, ricin toxin and doxorubicin. Without harming the spectators' cells, these multifunctional MS2 VLPs could selectively kill target cells. Francis *et al.* used orthogonal bioconjugation strategies to label MS2 on cages inside and outside with different functional molecules [116,117]. They have demonstrated chemistry aimed at reactive amines or tyrosine's selected presentation of the outer and fluorescein targeting and masking molecules as a small interior model molecule. Doxorubicin encapsulation at the core of the MS2 compartment was an alternative approach for improving drug delivery to target cells. It has been shown that decreased intracellular accumulation of doxorubicin in human hepatocellular carcinoma cells (Hep3B) due to the P-glycoprotein levels could be achieved by loading empty MS2 virus capsids using drug molecules. Since targeted, therapeutically loaded viral particles are internalized through receptor-mediated endocytosis, they can overcome P-glycoproteins' efflux mechanism and destroy cancer cells at lower drug levels (20-times enhancement) in comparison with free drugs. In addition, doxorubicin encapsulation inside the MS2 viral capsid showed a substantially different time-dependent cytotoxicity [118]. Similar findings were reported in RCNMV, CMV and HCRSV-specific doxorubicin-loaded cancer-targeted particles, which reduced the drug's cytotoxicity in nontarget cells due to cell uptake in target cells [119–121].

For a wide range of small molecular conjugations, rod-shaped VLPs are used as scaffolds. The benefit of rod-shaped viruses is to be constructed from more native virus protein subunits. TMV, for example, comprises 2130 primary coat-protein subunits, while the T_1/4_7 bacterio-figuration P22 is composed of only 420 subunits, one of the larger spherical capsids mentioned here. While rod-shaped viruses have less interior space per subunit, the sheer number of subunits is directly associated with the VLPs in more indigenous sites. The covalent binding of Gd chelates both inside and out of the TMV VLPs and in a thermally induced disordered TMV spherical particle morphology resulted in some of the highest loading factors estimated the small-molecule MRI in a VLP [122,123]. During 5 days, cucumber mosaic virus (CMV) drug carriers had a lasting drug release profile *in vitro*. In tumor-bearing animal models, the effectiveness of viral particles filled with doxorubicin was also reported. Treatment demonstrated a substantial delay in the development of tumors and improved survival with doxorubicin-loaded TMV particles due to a compelling accumulation of the nanomaterials within the tumor cells, while free doxorubicin had little impact on tumor size or survival in an animal model [124,125].

The infusion technique depends on the fact that viral particles swell so that pores are exposed in the capsid to disperse individual loads. The swelling then reversed allows the pores to close and the content to be stuck within. The mechanism for such reversible swelling of TBSV (the Tombusviridae) in tomato bushy stunt virus is based on chelation and added divalent cations that allow pores to be opened and closed. This process was used to load ethidium bromide TBSV virions. Besides, the chemical medicinal treatment doxorubicin has been used for loading into the red clover virus (RCNMV) necrotic mosaic virus by the association between the medicament and viral nucleic acid (VNA) [126]. The same technique was employed to load doxorubicin onto particles from the distantly associated cucumber mosaic virus (CMV [Bromoviridae]). This methodology is also used in agriculture when RCNMV's soil mobility is loaded into viral particles by the virus nematicide Abamectin [127,128].

The caging technique depends on the assembly *in vivo* or the disassembly *in vitro* of viral particles around the relevant payload. CMV is almost definitely the plant virus most widely used for caging foreign cargo. The suitable conditions have been identified as the conditions necessary to dismantle the virus particles and had a quite primary benefit: improvements in pH and ionic intensity rendered it easier to manage swelling and ultimately complete dismantling of the viral portion. This method's reversibility has allowed later researchers to use disassembly–reassembling processes to extract natural nucleic acid from the particles' inside and substitute it with Vargason of interest. This procedure has been studied extensively [129,130].

In one case, CCMV particles were used as vehicles for regenerative medicine: after disassembling the viruses, the native viral RNA was extracted, and dimers of CCMV were arranged around heterologous RNA sindbis mammalian virus. The researchers show that after cells were transfected with chimeric viral particles, the heterologous RNA was released and expressed in mammalian cells. The caging technique and any selective genetic modification of the CCMV capsid protein can also be used for the unique packaging of proteins inside CCMV particles [59]. In particular, the CCMV capsid protein N-terminus may be merged into the leucine zip K-coil while the E is fused into the E protein cargo C-terminus. Coli, which allows noncovalent load attachment to dimers and VLPs self-assembly around proteins cargo. Successful delivery through body tissue is a big concern of pharmacology in addition to the biocompatibility of medicines. This is particularly true concerning anticancer medications, which differentiate cancerous from healthy cells since some cell-surface receptors break up more quickly and differentially. Therefore, anticancer medications are harmful to all cells and, therefore, also have significant adverse events [131,132].

Furthermore, the development of stimuli-responsive protein-based nanomaterials remains an attractive approach for the development of multifunctional nanocarriers. VLPs of *Macrobrachium rosenbergii* nodavirus (MrNVLPs) have been developed as a multifunctional drug delivery vehicle, whereas folic acid was chemically conjugated to the surface exposed lysine amino acid, while doxorubicin was internally loaded within the MrNVLPs via infusion approach. Authors reported sustained DOX release in a thermally responsive manner with an increase in drug release in response to an elevated temperature of 43°C on HT29 cells than CCD841CoN normal cells while reducing DOX cytotoxicity on the normal cells as they lack the overexpression of folate receptor [133,134].

## Functionalization of viral capsids

Throughout medicine, nanotechnology and material science, viruses and phage are used in various applications. Phages are among other applications used to treat bacterial antibiotic resistance infections, monitor the use of phage-display technology for possible medicines and supply medicines by VNPs [135]. Combining VNPs and moieties like metals, polymers or image diagnostics opens avenues for developing novel materials such as catalysts, biomimetics or selectively targeted imaging agents [65,136–138]. With all five of the M13's coat proteins accessible on phage display, some features on the phage surface are critical for achieving existing properties. Three different M13 coat proteins used for the combined imaging and delivery of prostate cancer were developed on a phage-based therapeutic platform. DOX was coupled with the main protein of the surface, while other coat proteins (p3 and p9) have been used respectively as a cancer-targeting agent for peptides and fluorophores [139]. Alternatively, the drug-loaded material may be modified with viruses for targeted applications rather than loading drug molecules onto viral particles. For instance, the folic acid-modified M13 phage was coupled with a DOX-loaded polymer (poly-(caprolactone-b-2-vinylpyridine); PCL–P2vP), to target cancer cells [140]. The phage particles allow multivalent target–receptor interaction and enhanced targeting by controlling a large area with positioning and orientation. In addition, *in vitro* experiments with human nasopharyngeal cells have shown a substantial increase in cellular uptake and selectivity than free medicine for DOX-loaded phage-coated polymer particles [141].

**Figure 7.**
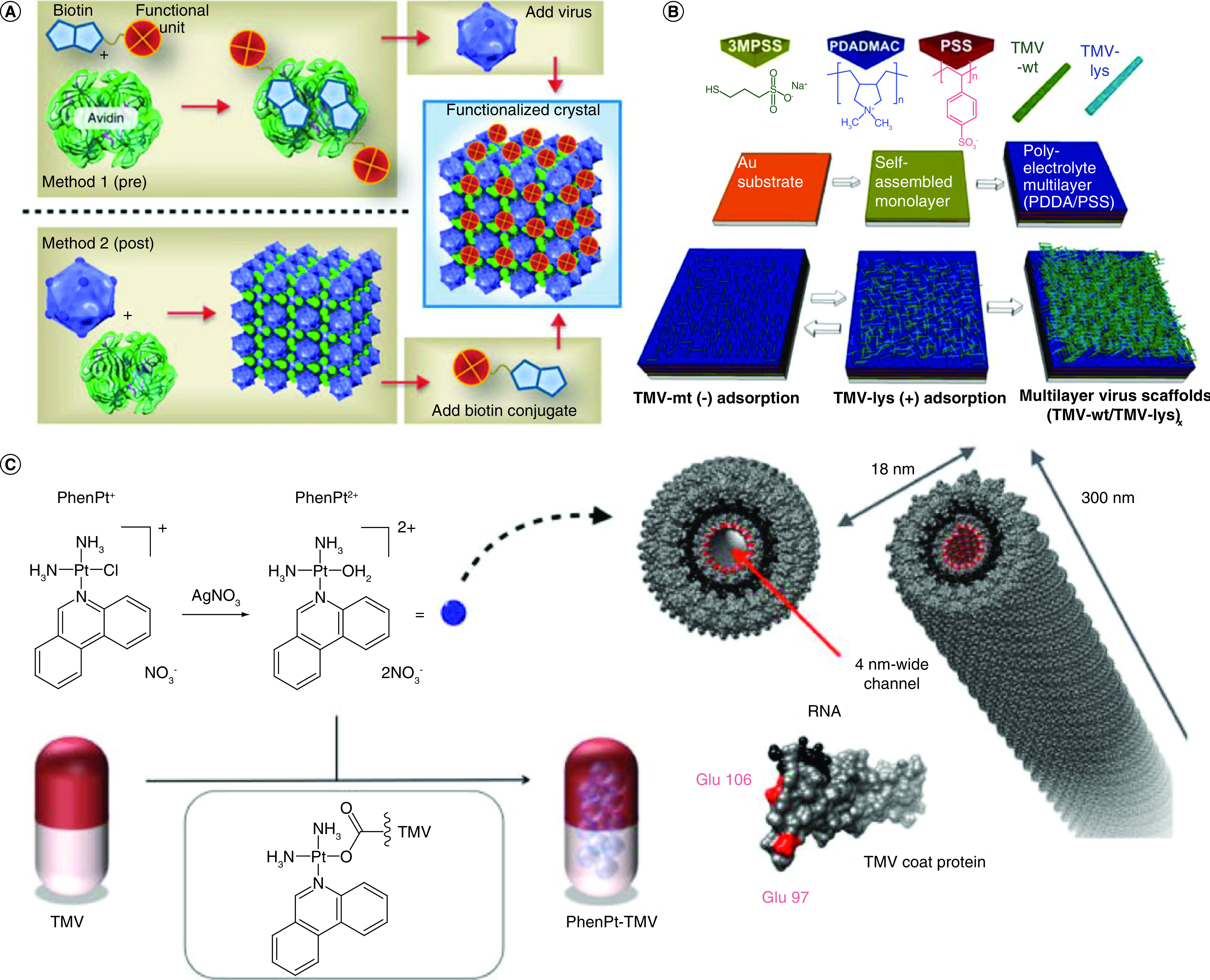
Capsid chemical engineering to impart new surface functionalities. **(A)** Functionalization approaches of congenital cytomegalovirus-avidin crystals [145]. **(B)** Sequential formation of multilayer virus scaffolds via layer-by-layer deposition of tobacco mosaic virus (TMV-wt) nanoparticles and mutant TMV particles with lysine residues (TMV-lys), Tiu *et al.* [147]. **(C)** Loading of phenanthriplatin into TMV [148]. TMV: Tobacco mosaic virus.

Decorative proteins are viral genetic accessories that decorate the surfaces of many phages and viral capsids. The capsid protein capable of enveloping anionic modules, such as heterologous RNAs, therapeutics, enzymes or gold nanoparticles, by loading the capsid's complementarity to the target cells by functioning the outside surface of the capsid. Similarly, CCMVs were filled with multiple moieties, including gold nanoparticles, negatively-loaded chromophores and polymers, from the closely related CPMV [142–144]. Liljeström *et al.* reported two methods in which electrostatically assembled CCMV (acidic)/avidin (basic) co-crystals can be functionalized selectively with biotin-tagged functional units [145,146]. Method 1: Prefunctionalization of avidin with the biotinylated functional unit followed by addition of the virus particles. Method 2: Assembly of the crystals followed by post functionalization with the biotinylated agent (Figure 7A). Similarly, Tiu *et al*.; proposed a bottom-up assembly of nanoscale building blocks for charged TMV through electrostatic layer-by-layer deposition of nanoparticles (Figure 7B) [147]. Czapar *et al.* proposed phenanthriplatin, *cis*-[Pt(NH_3_)_2_Cl(phenanthridine)](NO_3_), which is a cationic monofunctional DNA-binding platinum(II) anticancer drug candidate with unusual potency and cellular response profiles toward *in vivo* efficacy highlighting the need for a delivery system (Figure 7C) [148].

## Viral capsids & other nanomaterials as drug carriers

Functionalized and nonfunctionalized virus capsid have been established as a drug carrier in the field of medicine. However, their utility is specific to their safe utilization and the safe response of the body. Many other drug carriers have been established in the near past, which determined the different safety criteria. The majority of them belong to the class of nanomaterials. Various nanomaterials like gold nanoparticles, magnetic nanoparticles, quantum dots, organic materials like liposome-based materials, dendrimers, polymer and carbon-based nanoparticles such as carbon nanotubes, graphene and graphene oxides have been discovered for their utilization as a drug carrier [149–153]. The described nanomaterials can be categorized according to their chemistry; inorganic and organic materials as the base material. Organic materials include carriers based on polymers and lipids like dendrimers, liposomes, dendrimers and other polymer derivatives, while inorganic materials include materials like metal-based nanomaterials and carbon-based nanomaterials (fullerenes, graphene, graphene oxide) [154,155]. These nanomaterials are functionalized for their use as drug carriers, depending on the different physical and chemical parameters [156].

Though nanomaterials and virus capsid are in the establishment as a drug carrier, the utility and production depend on the physical parameters and chemistry. To use virus capsid, the isolation, purification and deployment require new technologies and methods and a lot of quality assessment to maximize extracted quantities; however, the synthetic and green method available for the nanomaterial's synthesis gives them the benefit ahead [89,144,157,158]. Discussing the pros and cons of using nanomaterials and viral capsid, the leverage of merits bends in the direction of the viral capsid, owing to being biological in origin. However, nanomaterials are being preferred due to their optimization capability.

## Conclusion

Over the last decades, dynamic viral structural science has gained significantly through technological advances in structure analysis, including achievements of cryo-EM and x-ray crystallography in the field of architectural biology. Many specifics reveal that many complex viruses, which harbor distant infections, have relatively similar strategies. As we understand, many viruses are evolving very differently over time. Adenovirus, which infiltrates mammals, uses shape, approaches and methodologies, striking similar to those utilized by the bacteriophage PRD1. The similarities between adenoviruses and PRD1 extend to their DNA replication pathways, capsid composition and the folding of their main proteins. More recently, PRD1 family members were identified or projected, and these lines now apply to major nucleocytoplasmic DNA viruses, including asfarvirus, iridovirus and the giant mimivirus viruses infecting bacteria or the archaea. All these viruses are composed of the very same sort of duplicate, pseudo-hexagonal capsomers, with a triangulation number ranging between T = 21 and T = 169, and up to 972< T <1200 for gigantic mimivirus, arranged in separate tiling structures. Strikingly, a non-icosahedral vaccinal protein folding like a pseudo-hexamer double-barrel reveals a potentially shared descendant of icosahedral virus dsDNA. Viruses have outstanding features and functionality. They have grown closely based on their live hosts and have an impact on life growth. The research of viral structures thus has implications over bioengineering. For example, viral molecules may be self-assembled into supramolecular systems, identify those goals and are highly adaptable to the environment.

## Future perspective

The development of novel biomaterials for advanced drug delivery is one of the current hot topic for the pharmaceutical industries and researchers working in drug delivery. We can envision that in the next 5 years, there could be several advances in the conventional drug delivery system, and it may not be a surprise that viral nanoparticles have been utilized as therapeutic carriers for selective drug targeting for inflammatory diseases and cancers, vaccine development, immunotherapy and molecular imaging.

**Executive summary**

- Viral nanoparticles have been utilized as therapeutic carriers for selective targeting.
- Viral capsids self-assemble through genetic or chemical modification for diverse clinical applications.
- Viral capsids are interesting scaffolds that could be engineered to impart new functionalities.
- The viral-based system is considered to be biodegradable, nontoxic and customizable therapeutic carriers.
